# Breeding Utilization of the White-Grained Pre-Harvest Sprouting-Resistant Wheat Landrace Tuotuomai Through Marker-Assisted Selection

**DOI:** 10.3390/plants15192974

**Published:** 2026-09-29

**Authors:** Xi Pu, Qinyun Liu, Huayu Jiang, Hao Tang, Huixue Dong, Xiaojiang Guo, Zhongwei Yuan, Mengping Cheng, Zhien Pu, Maolian Li, Wei Li, Qian Chen, Zehou Liu, Jun Li, Songtao Wang, Guoyue Chen, Wuyun Yang, Jirui Wang

**Affiliations:** 1 State Key Laboratory of Crop Gene Exploration and Utilization in Southwest China & Basic Research Zone for Crop Science in Sichuan Province, Sichuan Agricultural University, Chengdu 611130, China; 18483692553@163.com (X.P.); jhyu010202@163.com (H.J.); haotangsaas@163.com (H.T.); donghuixue@sicau.edu.cn (H.D.); bangzhanggxj@126.com (X.G.); yzwyvon2004@126.com (Z.Y.); qchen1120@163.com (Q.C.); gychen@sicau.edu.cn (G.C.); 2 Triticeae Research Institute, Sichuan Agricultural University, Chengdu 611130, China; 15309353132@163.com (Q.L.); mpcheng71250@163.com (M.C.); puzhien@sicau.edu.cn (Z.P.); 15281012179@163.com (M.L.); liw03@163.com (W.L.); 3 Crop Research Institute, Sichuan Academy of Agricultural Sciences, Chengdu 610066, China; zehouliu@163.com (Z.L.); lijunchd@126.com (J.L.); yangwuyun@126.com (W.Y.); 4 National Engineering Research Center of Solid-State Brewing, Luzhou 646000, China; wangst@lzlj.com

**Keywords:** seed dormancy, grain pigmentation, wheat breeding, molecular marker, KASP assay

## Abstract

Improving pre-harvest sprouting (PHS) resistance in white-grained wheat is challenging because reduced grain pigmentation is often associated with weaker seed dormancy. Here, we evaluated the breeding value of the white-grained, PHS-resistant landrace Tuotuomai (TTM) and developed a molecular marker for tracking TTM-derived material during breeding. Based on phenotypic segregation and sequence polymorphism analysis, a KASP marker targeting a polymorphic site in the *TaMyb10-D1* region was developed. The marker co-segregated with white grain color in the TTM × CS population, and the TTM-type marker genotype occurred more frequently in low-germination-percentage (GP) than in high-GP groups of the TTM × AK58 segregating populations. The marker was subsequently combined with phenotypic selection in pedigree and multi-parent breeding schemes. The derived white-grained line SMBK3 had a GP of 29.33%, compared with 98.67% for AK58, and showed reduced plant height and increased thousand-kernel weight relative to TTM. The multi-parent-derived line SMBK4 combined white grain with a GP of 46.93%, compared with 100% for the susceptible white-grained parents. Analysis of SMBK3-derived lines indicated that additional genetic factors, including the *PHS1* haplotype, also contributed to variation in GP. These results demonstrate the breeding value of TTM and support the use of the *TaMyb10-D1*-linked KASP marker as a tracking tool, together with phenotypic selection, in TTM-derived white-grained wheat breeding.

## 1. Introduction

Pre-harvest sprouting (PHS), defined as the early germination of freshly harvested mature seeds on the mother plants, severely reduces grain quality and yield in cereal crops [1]. Common wheat (*Triticum aestivum* L., AABBDD) feeds more than one-third of the global population [2] and is susceptible to PHS in most production regions, with estimated annual economic losses reaching $1 billion [3,4]. PHS not only reduces grain yield and weight but also markedly increases grain α-amylase activity, which degrades starch and lowers the falling number, thereby impairing flour quality, end-use processing, and seed vigor; sprouted grains are also more vulnerable to fungal contamination at harvest [5]. PHS is an increasing concern for wheat production, particularly as rainfall events around harvest become more frequent or unpredictable.

The correlation between grain color and seed dormancy/germination poses a major barrier to developing white-grained wheat with strong PHS resistance. Red-grained cultivars generally exhibit stronger resistance than white-grained ones [1,6,7]. Conversely, white wheat grains contain lower concentrations of phenolic compounds, resulting in reduced bitterness and a milder flavor profile, attributes that contribute to their preference in Asian markets [8]. Genetically, wheat grain color is determined by the R2R3-MYB transcription factors of *TaMyb10 (TaMyb10-A1*/*B1*/*D1*), which control flavonoid and proanthocyanidin accumulation in the pericarp and testa [6,9,10,11]. Notably, *TaMyb10-D* directly promotes ABA biosynthesis by transcriptionally activating *NCED*, thereby enhancing seed dormancy and PHS resistance [6]. The Huang–Huai Plain, China’s core wheat production belt, is dominated by white-grained commercial varieties that universally exhibit weak sprouting resistance. The vulnerability of white-grained wheat to harvest-time rainfall was highlighted by prolonged rainfall during wheat maturity in the Huang–Huai production region in 2023, which caused extensive sprouting and deterioration of harvested grain quality [12]. Breeding PHS-resistant white-grained wheat also faces several practical constraints. Strong PHS resistance is uncommon in elite white-grained germplasm, while field-based phenotypic screening is labor-intensive and sensitive to environmental variation. In addition, the use of resistant landrace donors may introduce undesirable agronomic traits that require several generations of selection to remove.

PHS resistance is controlled by multiple genetic and physiological mechanisms, with seed dormancy playing a central role. Major regulators include genes involved in ABA biosynthesis and signaling, such as *TaMFT*/*TaPHS1*, *Vp-1*, *TaMKK3-A*, and *TaSdr*, together with other factors affecting α-amylase activity and spike morphology [13,14,15,16]. Recent studies have further identified transcriptional, epigenetic, protein interaction, and calcium-signaling components of dormancy regulation, including *TaSRO1*, *TaGATA1-TaELF6-A1*, *PKL*, *TaPP2C-A6-TaDOG1L*, and *TaCNGC-2A* [13,17,18,19,20]. Consistent with this complex genetic architecture, PHS-associated QTLs have been detected across the wheat genome, with several major loci repeatedly identified on chromosomes 3A, 3B, 3D, and 4A [14,21]. These findings have facilitated the development of molecular markers for PHS-related loci, while genome editing of dormancy- and grain-color-related genes has provided additional strategies for improving PHS resistance [22,23,24,25].

Wheat landraces constitute a primary reservoir of PHS resistance alleles, especially in regions with prolonged exposure to humid harvest conditions [26,27]. In China, several wheat landraces carry PHS-resistant loci that operate independently of grain color [26,28], such as the white-grained landrace Tuotuomai (TTM). The elite *TaMYB7-A1* haplotype introgressed from wild einkorn wheat confers PHS resistance independently of grain color and without yield penalty [29]. Other genetic resources have also been exploited, including *Aegilops tauschii*-derived *QDor.3D*, for which candidate genes and diagnostic KASP markers have been identified to facilitate marker-assisted selection [30]. Modern breeding has reduced part of the genetic diversity retained in wheat landraces, including potentially useful alleles for stress adaptation [31]. Therefore, exploring and utilizing PHS-resistant landraces is essential to broaden the narrow genetic basis of modern white-grained wheat breeding pools. Nevertheless, the practical breeding potential of TTM has not been systematically explored, and the lack of effective molecular markers to track its PHS resistance-associated locus in segregating populations has limited its use in breeding.

In this study, we evaluated the genetic and breeding characteristics of the white-grained PHS-resistant landrace TTM and developed a *TaMyb10-D1*-linked KASP marker for tracking TTM-derived material. We then examined the association of this marker with grain color and GP in segregating populations and applied it together with phenotypic selection in pedigree and multi-parent breeding schemes.

## 2. Results

### 2.1. TTM Provides a Breeding Source of Low GP in a White-Grained Background

**Figure 1:**
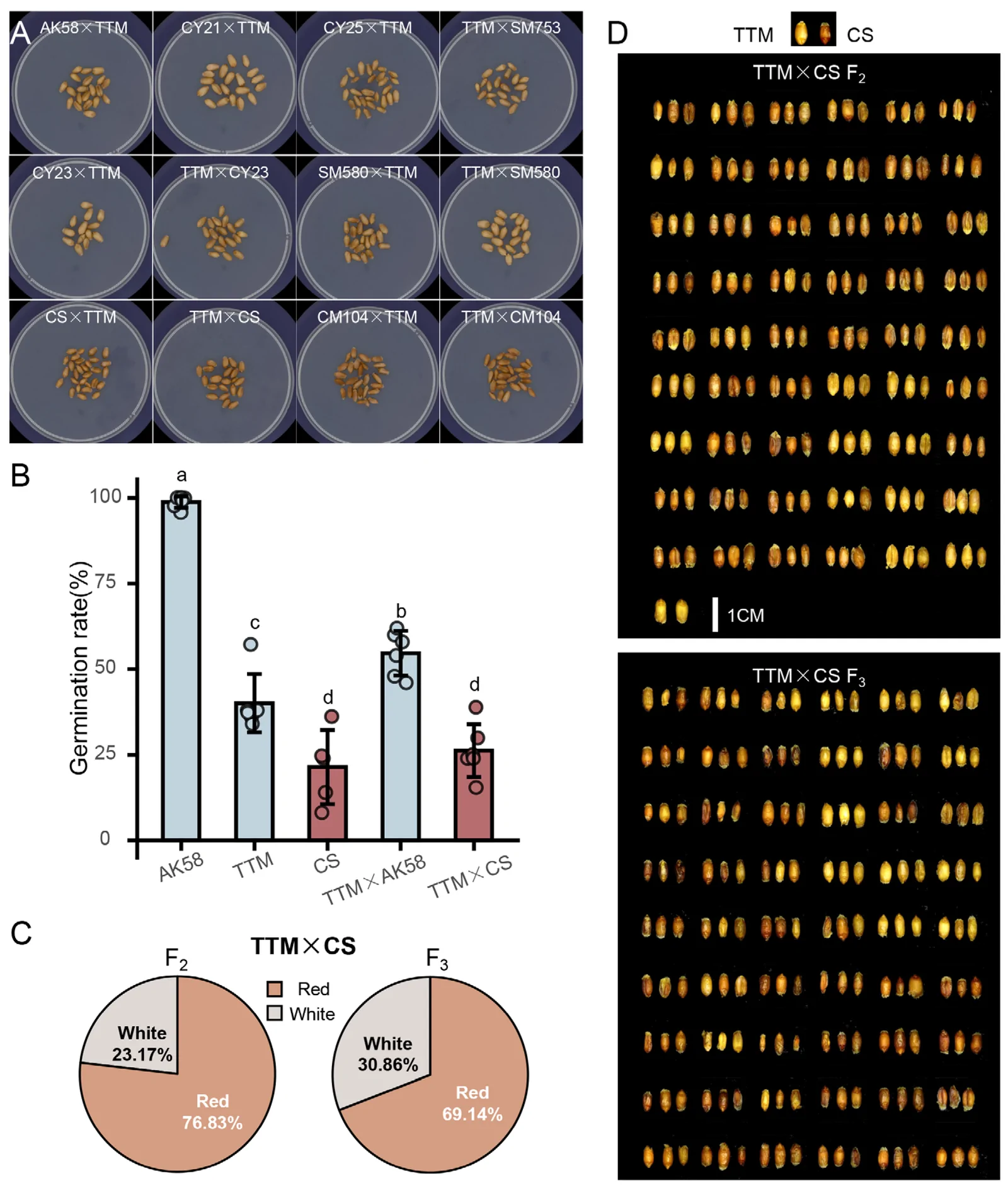
Phenotypic variation in grain color and germination percentage among Tuotuomai-derived populations. (**A**) Grain color phenotypes of F_1_ progenies from crosses between TTM and various white-grained (AK58, CY21, CY23, CY25, SM580, SM753) or red-grained (CS, CM104) parental lines. (**B**) Germination percentages of AK58, TTM, CS, TTM × AK58 F_1_, and TTM × CS F_1_. Error bars indicate SD. Different letters denote significant differences at *p* < 0.05 according to Tukey’s HSD test. (**C**) Segregation ratios of grain color in the F_2_ and F_3_ generations of TTM × CS. (**D**) Grain color identification in the TTM × CS population after NaOH soaking. Red-grained lines are marked with red dots, and white-grained lines with white dots.

TTM was crossed with seven white-grained (AK58, CY21, CY23, CY25, SM580, SM753, and MY1848) and three red-grained (CS, CM104, and AS661634) wheat lines to examine grain-color inheritance and germination responses (Table 1). All F_1_ progeny from crosses between TTM and white-grained parents had white grains, whereas those from crosses with red-grained parents had red grains. Reciprocal crosses (e.g., TTM × CS vs. CS × TTM) produced identical grain-color phenotypes, confirming nuclear inheritance (Figure 1A; Table A1). This indicates that red grain color is dominant over white and that the white phenotype of TTM is controlled by recessive allele(s). To further investigate the genetic control of grain color, we developed and characterized segregating populations. In the TTM × CS cross, the observed segregation ratio of red to white grains in the F_2_ population fit a 3:1 Mendelian segregation ratio, which was further corroborated by the segregation pattern in F_3_ families (Figure 1C,D; Table A2). This indicated that a single major recessive locus governed white grain formation in the CS background. In contrast, the F_2_ population derived from TTM × AS661634 deviated from the expected monogenic 3:1 ratio but fit a 15:1 digenic ratio (Table A2). Thus, white grain inheritance from TTM is background-dependent.

The genetic basis of grain germination was analyzed by crossing TTM with the PHS-resistant parent CS and the PHS-susceptible parent AK58. The F_1_ progeny from TTM × CS showed a GP comparable to that of CS. In contrast, the F_1_ progeny from TTM × AK58 showed an intermediate GP, lower than that of AK58 but higher than that of TTM (Figure 1B). The intermediate F_1_ phenotype indicated that low GP in TTM was not inherited as a simple dominant trait in the AK58 background. Partial dominance or additive effects of dormancy-related loci have been reported in other wheat populations [3,15], consistent with the intermediate GP observed here.

### 2.2. A TaMyb10-D1-Linked KASP Marker Enables Tracking of TTM-Derived Material

**Figure 2:**
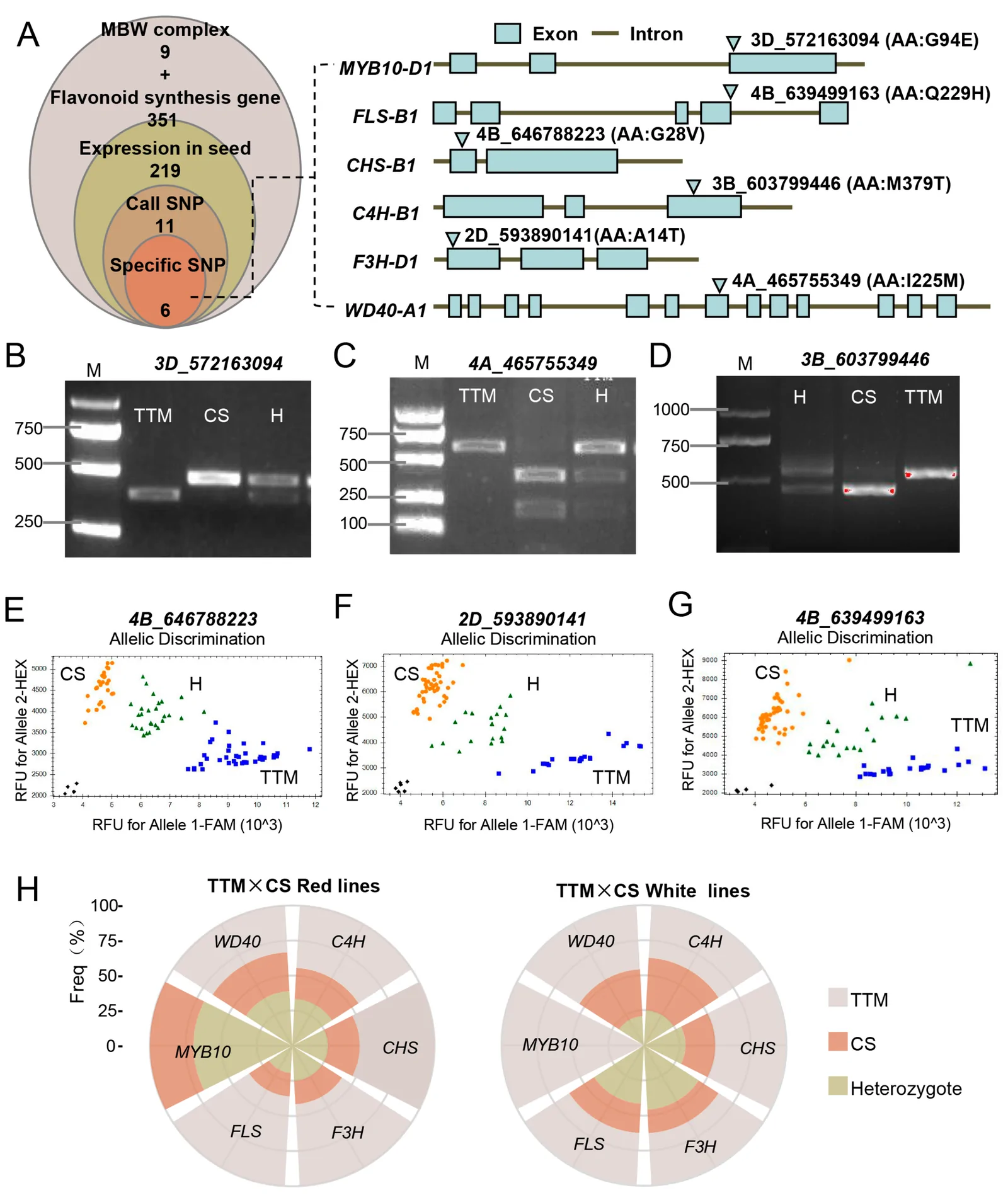
Development and genetic validation of a *TaMyb10-D1*-linked marker in TTM-derived populations. (**A**) Sequence polymorphisms selected for molecular marker development based on TTM whole-genome resequencing data. Six polymorphic sites used for marker development are indicated by arrows. (**B**–**G**) CAPS markers and KASP markers developed for the variants. (**H**) Distribution of marker genotypes in the TTM × CS F_2_ population, showing complete co-segregation between the TTM-type marker genotype and the white-grain phenotype.

**Figure A1:**
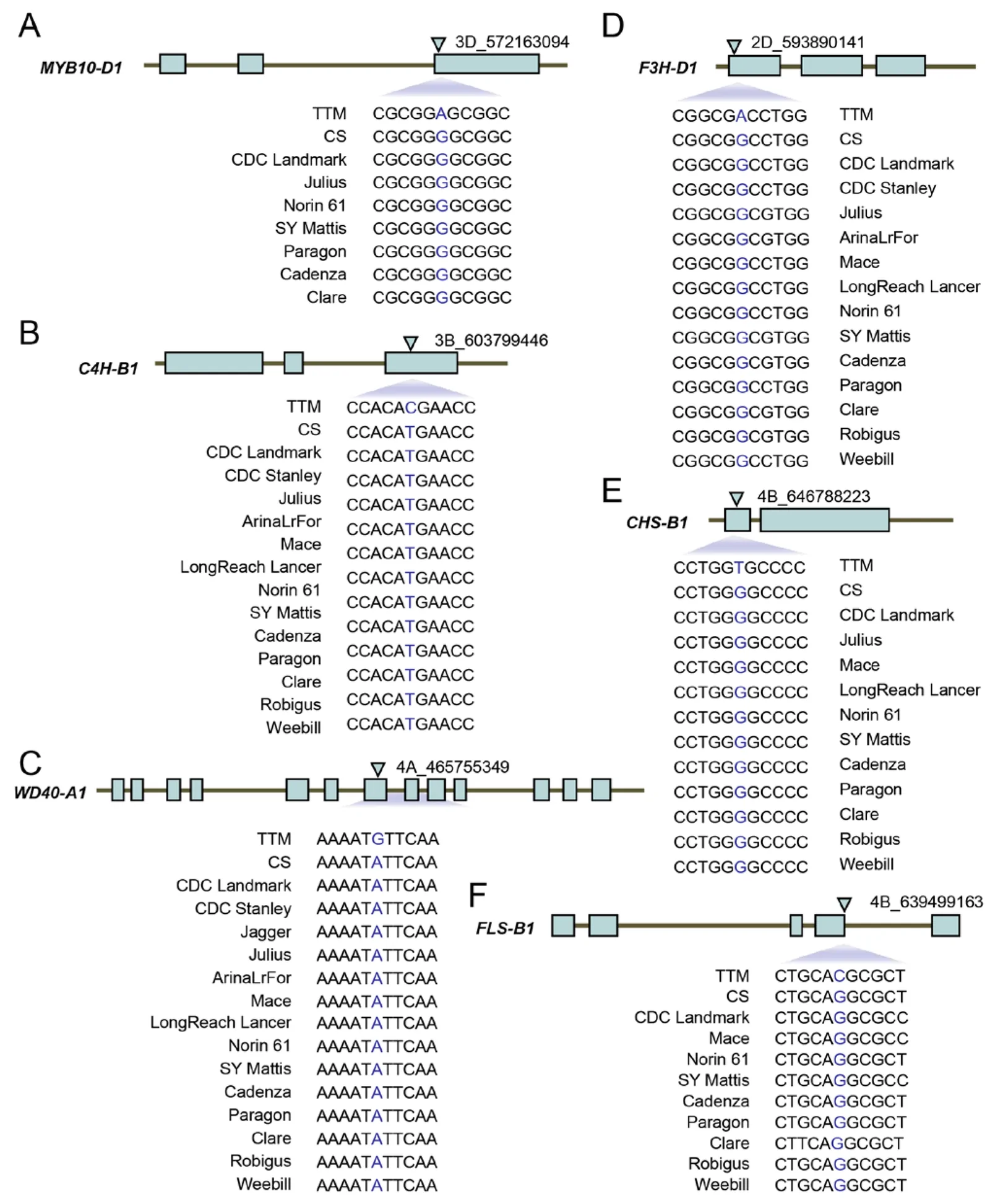
Sequence comparison of polymorphic sites selected for molecular marker development across wheat reference genomes. (**A**–**F**) Sequence polymorphisms selected for marker development in *Myb10-D1* (**A**), *C4H-B1* (**B**), *WD40-A1* (**C**), *F3H-D1* (**D**), *CHS-B1* (**E**), and *FLS-B1* (**F**) are indicated by arrows. Variations were identified through alignment with wheat 10+ reference genomes. Some reference genomes are absent from the alignment of individual candidate genes because their assemblies lack the relevant gene sequences.

To develop molecular markers for tracking TTM-derived material in breeding populations, sequence polymorphisms in genes involved in flavonoid biosynthesis and regulation were screened using the TTM resequencing data. Six polymorphic sites were selected for marker development (Figure A1 and Figure 2A; Table A3). CAPS or KASP assays were developed for these sites and tested for linkage with grain color in the TTM × CS F_2_ population (Figure 2B–G; Table A4). Genetic linkage analysis showed that only the marker targeting SNP 3D_572163094 was linked to grain color, with an LOD score > 45. This SNP is located within the *TaMyb10-D1* region, previously associated with grain color and PHS-related traits [6,7]. The TTM-type marker genotype completely co-segregated with the white-grain phenotype in the TTM × CS F_2_ population (χ^2^ = 64.0, *p* < 0.0001), whereas markers targeting the other five polymorphic sites segregated independently (Figure 2H; Table A5). The *TaMyb10-D1*-linked marker was therefore selected for subsequent evaluation in TTM-derived breeding populations.

**Figure A2:**
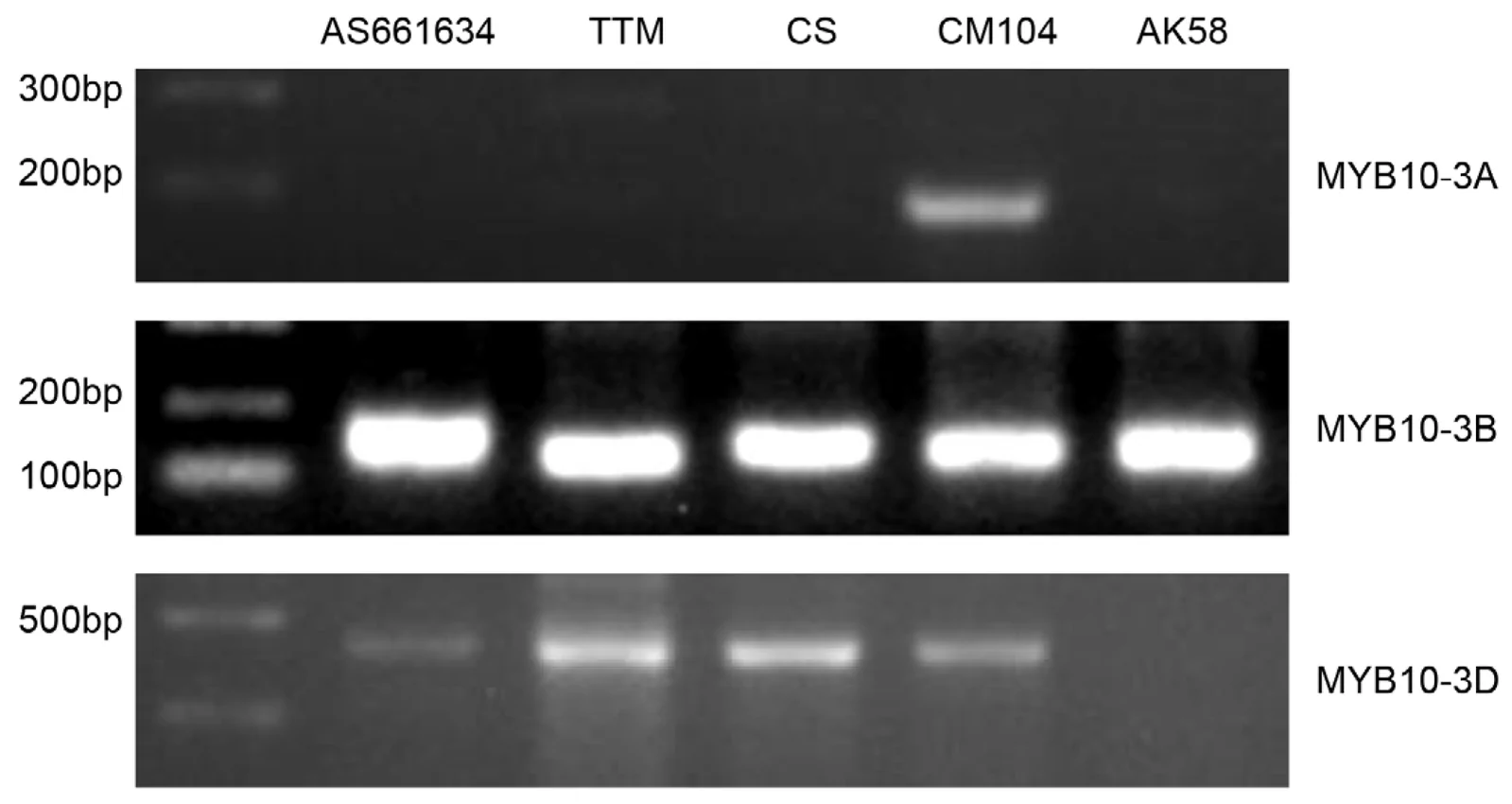
Genotyping results of the *TaMyb10-A1*, *TaMyb10-B1*, and *TaMyb10-D1* genes in different materials. The functional *TaMyb10-A1* allele yields a 268 bp specific band, while the loss-of-function variant (*TaMyb10-A-III*^Dele^) produces no amplification product due to a sequence deletion. The functional *TaMyb10-B1* allele yields a 161 bp specific band, whereas the loss-of-function variant (*TaMyb10-B-IV*^Dele^), which carries a 19 bp deletion, produces a 142 bp band. The functional *TaMyb10-D1* allele yields a 479 bp specific band, and the loss-of-function variant (*TaMyb10-D-V*^Dele^) produces no amplification product due to full locus deletion.

To evaluate how the recipient genetic background affects the relationship between the marker and grain color, we further genotyped the seven white-grained individuals from the TTM × AS661634 F_2_ population. All seven individuals carried the homozygous TTM-type marker genotype. Genotyping further showed that AS661634 harbored functional alleles [7] at *TaMyb10-B1* and *TaMyb10-D1* but a loss-of-function allele at *TaMyb10-A1*, whereas both TTM and CS harbored loss-of-function alleles at *TaMyb10-A1* and *TaMyb10-B1* (Figure A2). Thus, grain color in the TTM × AS661634 population segregated at two loci, *TaMyb10-B1* and *TaMyb10-D1*, resulting in the observed 15:1 (red:white) F_2_ ratio, as the white-grain phenotype required homozygous recessive alleles at both loci. These results indicate that the relationship between the *TaMyb10-D1*-linked marker and grain color depends on the *TaMyb10* genotype of the recipient parent.

**Figure 3:**
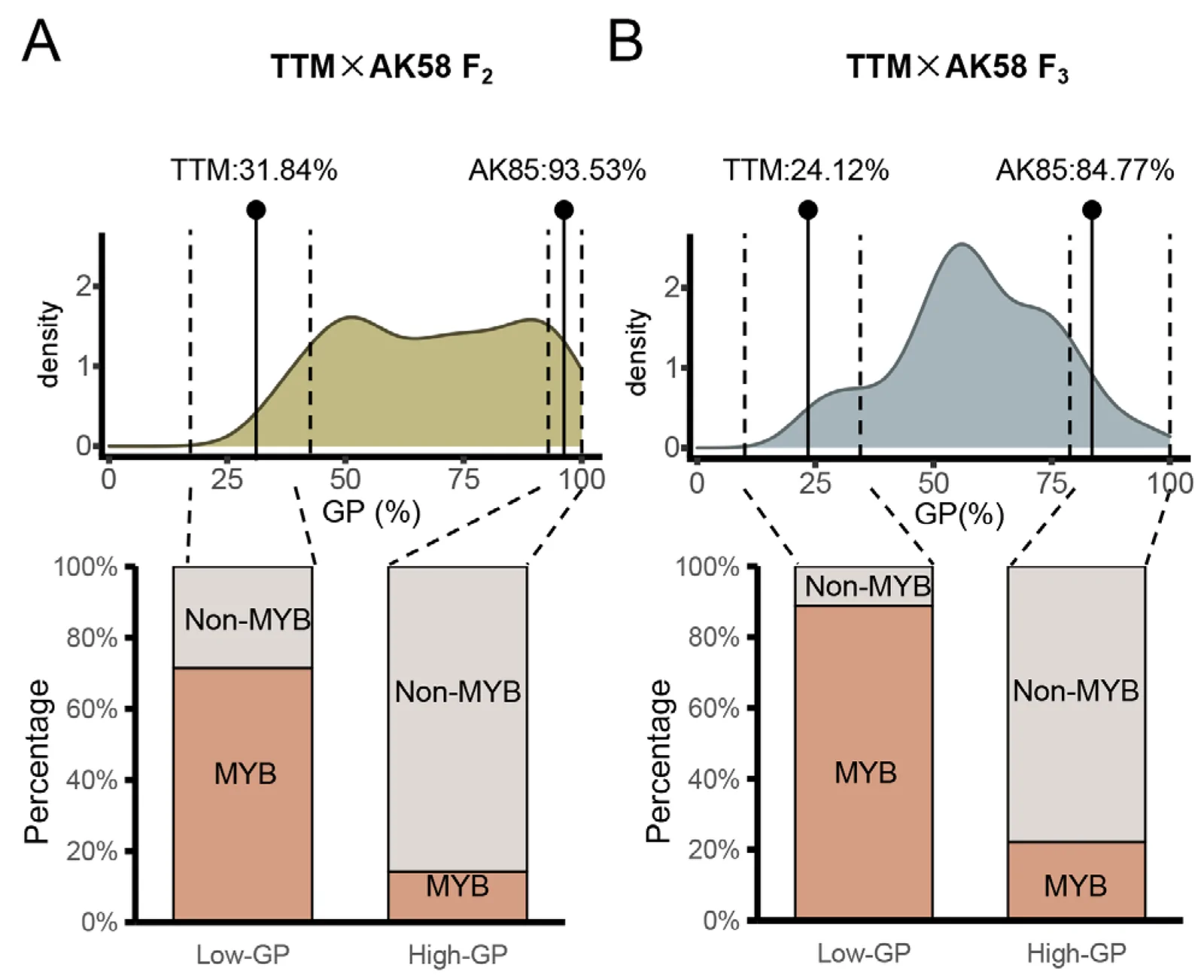
Association of the *TaMyb10-D1*-linked marker with germination percentage in TTM × AK58 populations. (**A**,**B**) Frequencies of the TTM-type marker genotype in low-GP and high-GP groups selected from the F_2_ (**A**) and F_3_ (**B**) generations of the TTM × AK58 population. Black dashed lines indicate the GP values of the parental lines evaluated in the corresponding year. The vertical dotted lines represent the GP thresholds for classification into low-GP and high-GP groups.

We next evaluated whether the *TaMyb10-D1*-linked marker could also assist selection for lower GP in a white-grained breeding population. TTM was crossed with the susceptible white-grained cultivar AK58, and marker–trait association was evaluated in low-GP and high-GP groups from the segregating populations. The TTM-type marker genotype occurred more frequently in the low-GP group than in the high-GP group in both generations. In the F_2_ generation, its frequency was 71.43% in the low-GP group compared with 14.29% in the high-GP group, although the difference was not significant (Fisher’s exact test, *p* = 0.1026). In the F_3_ generation, the corresponding frequencies were 88.89% and 22.22%, respectively, with a significant difference between the two groups (*p* = 0.0152; Figure 3A,B). Overall, the *TaMyb10-D1*-linked marker showed an association with lower GP in the evaluated TTM × AK58 populations and was subsequently used to track TTM-derived material during breeding.

### 2.3. Marker-Assisted Pedigree Breeding Improves the Agronomic Background of TTM-Derived Resistance

**Figure 4:**
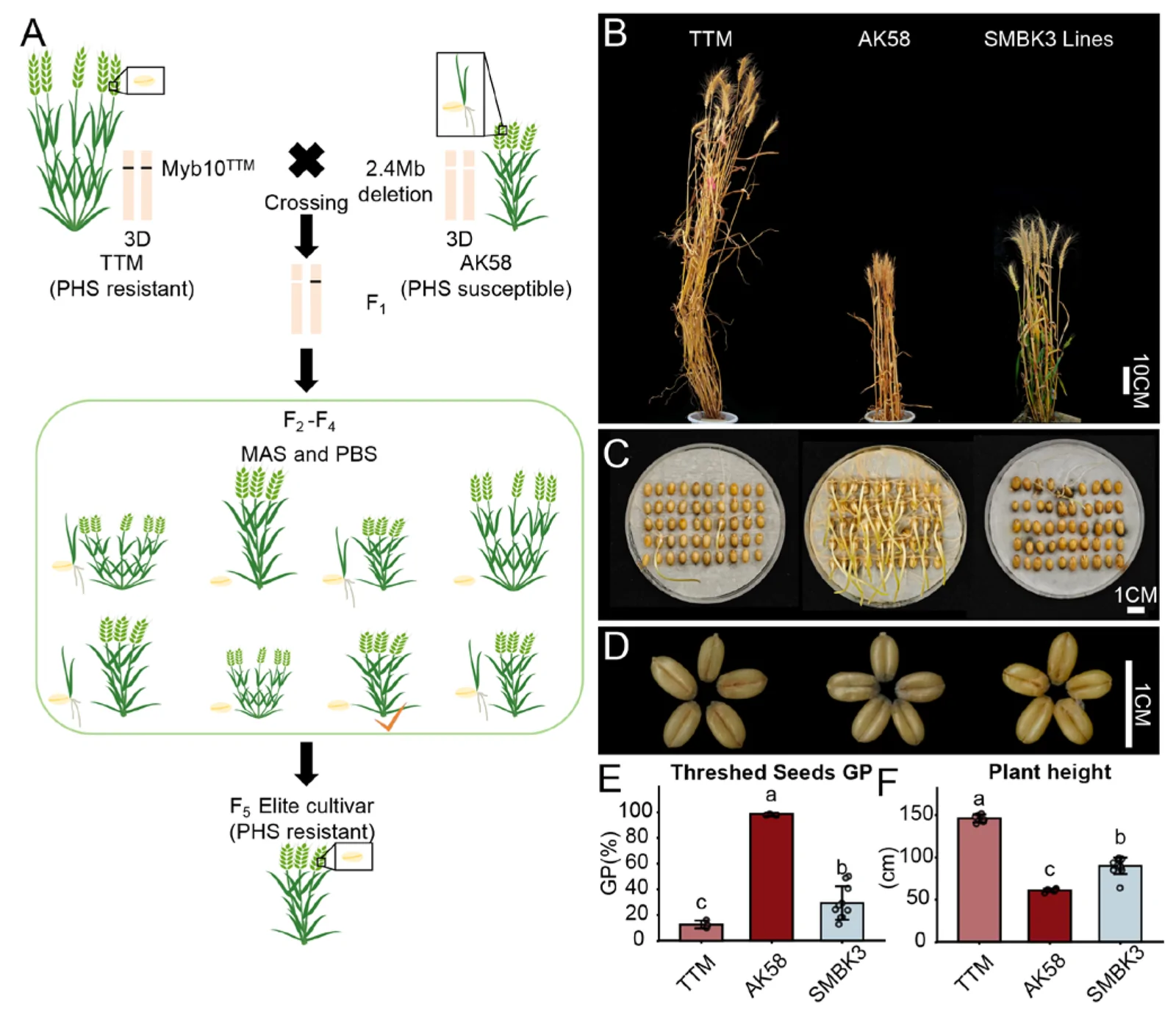
Marker-assisted introgression of TTM-derived material through pedigree breeding. (**A**) Scheme for developing new wheat germplasms by pedigree breeding. (**B**) Whole-plant morphology of TTM, AK58, and SMBK3 at maturity. (**C**) Seed germination phenotype of the three wheat lines. (**D**) Grain morphology of the three wheat lines. (**E**,**F**) Germination percentages (GP) (**E**) and plant height (**F**) of the three wheat lines. Error bars indicate SD. Different letters denote significant differences at *p* < 0.05 according to Tukey’s HSD test.

**Figure 5:**
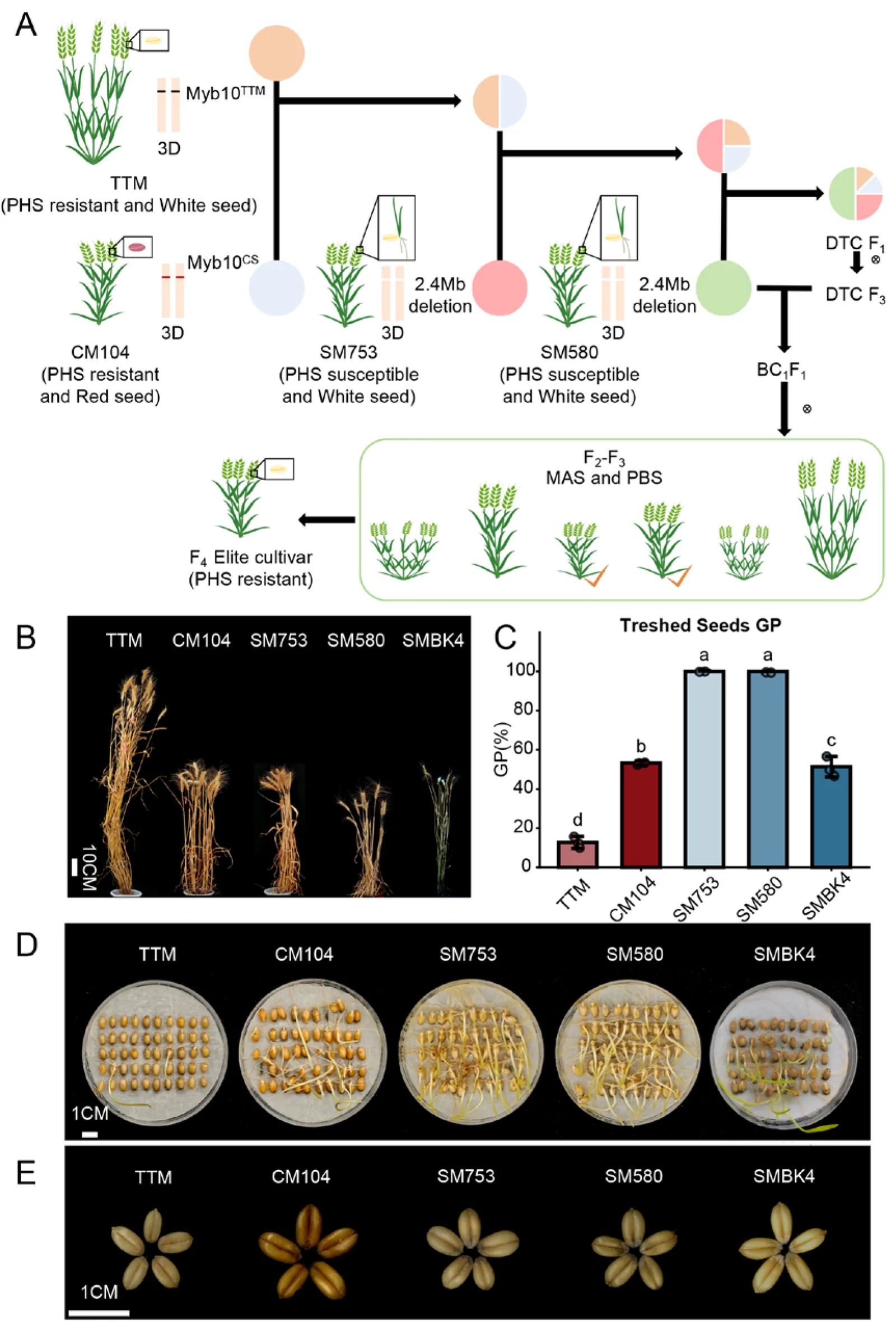
Application of the *TaMyb10-D1*-linked marker in multi-parent breeding. (**A**) Scheme for developing new wheat germplasms by DTC, The asterisk symbol (⊗) represents self-pollination. (**B**) Whole-plant morphology of TTM, CM104, SM753, SM580, and SMBK4 at maturity. (**C**) Germination percentages (GP) of threshed seeds from the five wheat lines. (**D**) Seed germination phenotype of threshed seeds from the five wheat lines. (**E**) Grain morphology of mature seeds from the five wheat lines. Error bars indicate SD. Different letters denote significant differences at *p* < 0.05 according to Tukey’s HSD test.

Landrace TTM has several agronomic characteristics that limit its direct use in modern wheat production, including tall stature (135 cm), loose plant architecture, and a relatively low thousand-kernel weight (TKW, 40.67 g). To introduce TTM-derived PHS resistance into modern white-grained wheat, we developed several crosses between TTM and modern cultivars and used the *TaMyb10-D1*-linked marker to track TTM-derived material during selection (Figure 4A and Figure 5A).

**Figure A3:**
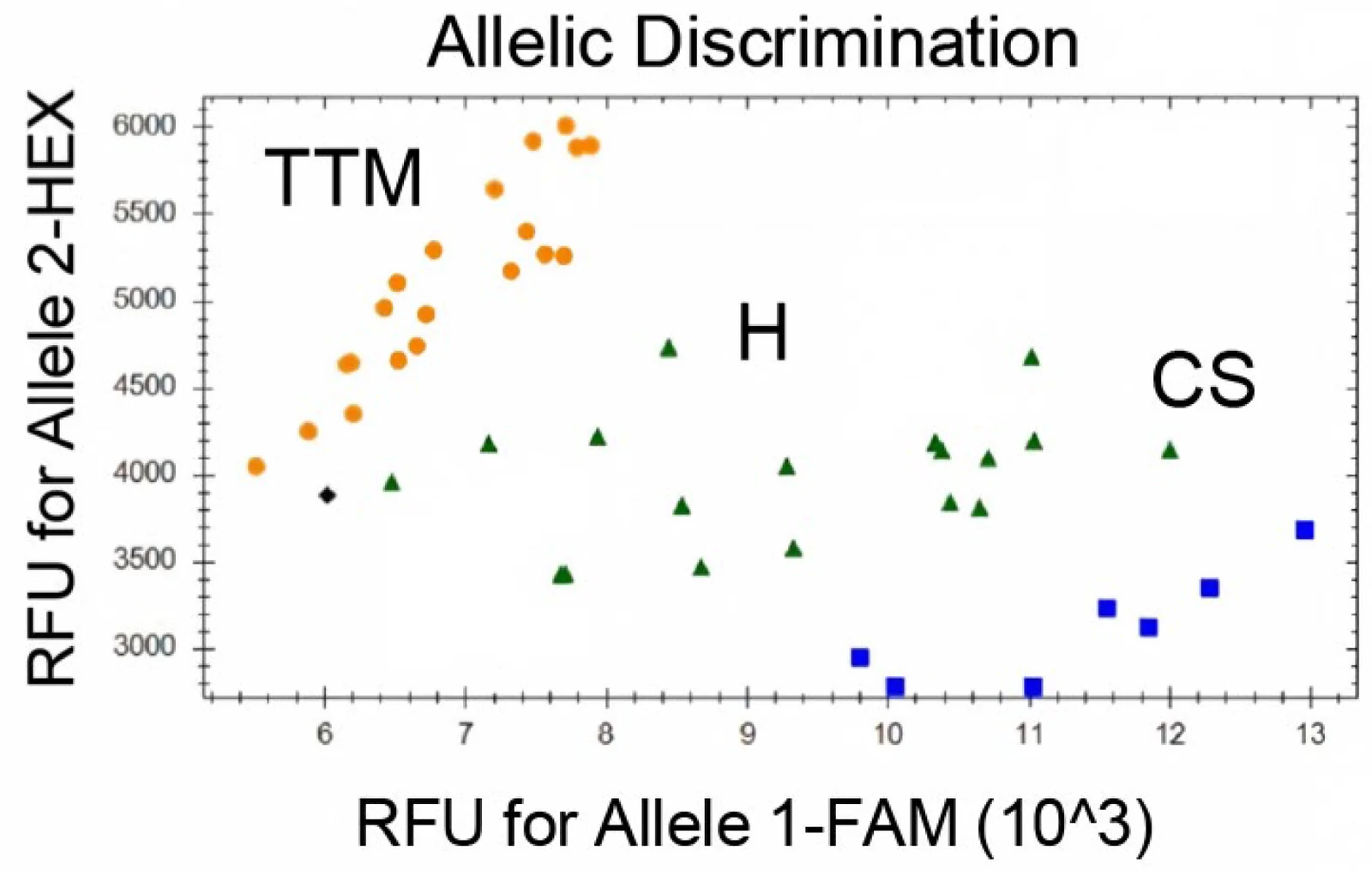
Allelic discrimination at the *TaMyb10-D1* locus using a KASP marker. The plot shows genotypes of individuals based on FAM (x-axis) and HEX (y-axis) fluorescence signals.

In the pedigree breeding scheme, the PHS-susceptible white-grained cultivar AK58 was used as the recipient parent. The KASP marker was applied from the F_2_ generation onward in combination with phenotypic selection (Figure A3; Table A4), using a marker-assisted selection strategy that has been successfully applied to PHS resistance in wheat breeding programs [24]. A stable line, SMBK3 (BK3), was obtained by the F_5_ generation. SMBK3 retained white grain color and had a GP of 29.33%, compared with 98.67% for AK58 (Figure 4C–E; Table A6). SMBK3 also differed from TTM in several agronomic traits: plant height was reduced from 135 to 89 cm, and TKW increased from 40.67 to 49.44 g (Table A6). Among the SMBK3 F_5_ lines, the lowest mean GP (27.43%) was observed in lines carrying both the TTM-type *TaMyb10-D1* marker genotype and the resistant *PHS1* haplotype [15], whereas lines lacking both marker haplotypes had a mean GP of 52.87% (Table A7). Although some genotype classes contained very few lines, the observed GP variation among SMBK3-derived lines suggests that the *TaMyb10-D1*-linked marker alone does not account for the low-GP phenotype.

### 2.4. Multi-Parent Breeding Combines TTM-Derived Low GP with Complementary Elite Traits

The marker was also tested in a multi-parent DTC (double top cross) breeding scheme involving CM104, SM753, SM580, and TTM (Figure 5A) [32]. The parental lines contributed complementary agronomic characteristics, including improved stripe rust resistance from CM104, stripe rust resistance and lodging tolerance from SM753, and white grain and early maturity from the recurrent parent SM580. The *TaMyb10-D1*-linked marker was used to track TTM-derived material during selection, and an advanced white-grained line, SMBK4 (4767), was obtained by the F_4_ generation. Line SMBK4 showed reduced GP and improved plant height relative to TTM (Figure 5; Table A6). The GP of line SMBK4 was 46.93%, compared with 100% for the susceptible white-grained parents SM580 and SM753 and 53.33% for the red-grained parent CM104 (Figure 5C–E; Table A6).

## 3. Discussion

### 3.1. Wheat Landraces Remain Valuable Resources for Improving PHS Resistance

TTM combines relatively low GP with white grain, but its tall stature and relatively low TKW necessitate the transfer of this phenotype into adapted breeding backgrounds (Figure 4B; Table A6). Modern breeding has reduced part of the diversity retained in landrace germplasm [33,34], and the SMBK3 and SMBK4 lines demonstrate that TTM-derived low GP can be retained while improving plant architecture.

### 3.2. Marker-Assisted Selection Facilitates the Utilization of TTM in Breeding

Improving PHS resistance in white-grained wheat is challenging because grain pigmentation and seed dormancy are genetically associated through the flavonoid regulatory pathway [35,36]. In the populations examined here, the *TaMyb10-D1*-linked marker co-segregated with white grain and was associated with lower GP, providing a means of tracking TTM-derived material during breeding (Figure 2H and Figure 3A,B). The KASP marker was subsequently applied together with phenotypic selection in both pedigree and multi-parent breeding schemes [24,37]. SMBK3 and SMBK4 retained the TTM-type marker genotype and had lower GP than the corresponding susceptible white-grained parents (Figure 4 and Figure 5). In practice, the marker was most useful as a complement to phenotypic selection rather than as an independent predictor of PHS resistance. Selection for the TTM-type marker genotype retained the targeted donor segment, whereas direct phenotypic selection remained necessary for GP and agronomic performance.

### 3.3. The Breeding Value and Limitations of the TaMyb10-D1-Linked Marker

Previous germplasm analysis indicated that PHS resistance is not common among the commercial white-grained wheat accessions examined [26,38]. The *TaMyb10-D1*-linked KASP marker was useful for tracking TTM-derived material in the populations examined here. However, the marker has several limitations. Its relationship with grain color depends on the *TaMyb10* genotype of the recipient parent, as demonstrated by the different segregation patterns in the TTM × CS and TTM × AS661634 populations (Figure A2; Table A2). More importantly, the association between the marker and lower GP was incomplete, indicating that additional genetic factors contribute to PHS resistance in TTM-derived populations.

In the SMBK3-derived population, lines with very low GP carried both the TTM-type *TaMyb10-D1* marker genotype and the resistant *PHS1* haplotype (Table A7). The reduced GP of these materials therefore likely reflects the combined contribution of multiple loci rather than the *TaMyb10-D1*-linked region alone. Combining the TTM-derived marker with markers for independent dormancy loci may consequently provide a more effective strategy for improving PHS resistance [15].

The marker alone is also insufficient for recovering an adapted agronomic background. Successive generations of marker-assisted and phenotypic selection reduced plant height and increased TKW relative to TTM, but SMBK3 and SMBK4 still differed from modern cultivars in some yield-related traits (Table A6). These residual differences may reflect linked donor segments or other characteristics inherited from the landrace background. Further crossing and selection will therefore be required to retain the desired TTM-derived traits while improving the agronomic background [39].

### 3.4. Future Perspectives for Genetic Dissection of PHS Resistance in TTM

The *TaMyb10-D1*-linked KASP marker developed in this study can be used to track TTM-derived material in the breeding populations examined here. The marker should presently be regarded as a breeding marker for the TTM-derived *TaMyb10-D1* region rather than as an independent predictor of PHS resistance. PHS-related QTL have previously been reported on chromosome 3DL, including regions near the *TaMyb10-D1* locus [26,40], but most have not been resolved to individual genes. Whether the association observed here reflects *TaMyb10-D1* itself or another locus within the *TaMyb10-D1*-linked region remains unresolved. Further genetic analysis using segregating populations will be required to determine the genetic basis of PHS resistance in TTM. QTL mapping followed by fine mapping and functional analysis could establish whether the *TaMyb10-D1*-linked region contributes directly to the phenotype and could identify additional loci underlying the low GP of TTM. Resolving these loci would also enable the development of more specific markers for retaining PHS resistance while reducing unwanted donor segments during breeding.

## 4. Materials and Methods

### 4.1. Plant Materials

TTM was collected from Suining Sichuan Province, China. For genetic analysis, TTM was crossed with multiple red-grained cultivars, including Chinese Spring (CS), Chuanmai 104 (CM104), and AS661634, as well as white-grained cultivars, including Aikang 58 (AK58), Chuanyu21 (CY21), Chuanyu23 (CY23), Chuanyu25 (CY25), Shumai580 (SM580), Shumai753 (SM753), and Mianyang1848 (MY1848). Primary segregating populations were developed for specific analyses: (i) an F_2_ population and derived F_3_ families from the TTM × CS cross were used for Mendelian inheritance analysis; (ii) an F_2_ population from the TTM × AS661634 cross was used to study background-dependent inheritance; (iii) F_2_ and F_3_ populations from the TTM × AK58 cross were used to evaluate the association between the *TaMyb10-D1*-linked marker and GP. All plants were grown in Wenjiang (30°42′18.9″ N, 103°49′46.1″ E) or Chongzhou (30°36′27.02″ N, 103°37′4.10″ E), Sichuan, China.

### 4.2. Phenotyping

For germination assays, grains were sampled at physiological maturity (approximately 35–40 days after anthesis) from the parental lines, F_1_ hybrids, segregating populations, and advanced breeding lines used in this study (Figure 1; Table 1 and Table A6). Grains were air-dried for 7 days in a well-ventilated and dry environment at ambient temperature, then stored at −20 °C to maintain dormancy. For each line or population tested, three replicate Petri dishes, each containing 50 undamaged seeds, were used for the germination assay. Seeds were incubated on filter paper with 5 mL of distilled water at 20 °C in the dark for 7 days. GP was calculated for each replicate as the percentage of germinated seeds at 7 days after imbibition (DAI), and the three replicate values were used to calculate the mean and standard deviation. Grain color of parental lines was determined by visual inspection of intact grains under natural light by three independent observers, based on the color of the pericarp and testa layers. For genetic populations, grains were soaked in 5% NaOH and incubated at 50 °C for 30 min to enhance color visualization.

### 4.3. TTM Genome Resequencing and Data Analysis

Genomic DNA was extracted from approximately 100 mg of fresh young leaves of TTM seedlings using the CTAB method [41] and quantified via NanoDrop 2000 (Thermo Fisher Scientific, Waltham, MA, USA). Whole-genome resequencing was performed by BerryGenomics (Beijing, China), and sequencing reads were aligned to a panel of wheat reference genomes via BWA, including Chinese Spring (CS) v2.1 (IWGSC RefSeq v2.1), Norin 61 (PGSB v2.1 assembly), nine IWGSC pan-genome chromosome-scale assemblies (Cadenza, Paragon, Mace, ArinaLrFor, CDC Landmark, CDC Stanley, SY Mattis, Julius, LongReach Lancer), as well as two independent chromosome-scale de novo assemblies (Robigus and Weebill). BAM files were processed (sorted, duplicate-marked, locally realigned, and quality-recalibrated) with SAMtools v1.15 [42] and GATK v4.2.6.1 [43] to calculate coverage and depth. SNPs and InDels were called using BCFtools v1.15 [44].

### 4.4. Molecular Marker Development

CAPS markers were generated using dCAPS Finder 2.0 [45], and primers were designed via the Triticeae Multi-omics Center (http://202.194.139.32/ (accessed on 10 May 2023). KASP primers were designed using PrimerServer (accessed on 10 May 2023) [46] on the same platform. KASP assays were performed in 5 µL reactions containing 2.5 µL 2× KASP Master Mix (Jasongen, Nanjing, China), 0.14 µL KASP primer mix (12 µM of each allele-specific primer and 30 µM of the common primer), and 20 ng of template DNA, on a Bio-Rad CFX96 PCR instrument (Bio-Rad, Hercules, USA). Thermal cycling was performed as follows: 94 °C for 15 min; 10 cycles of 94 °C for 20 s and 61 °C for 60 s, with the annealing temperature dropping by 0.6 °C per cycle, followed by 32 cycles of 94 °C for 20 s and 55 °C for 60 s; endpoint fluorescence was read at 25 °C. Genotypes were scored from the FAM/HEX fluorescence signals using the CFX Manager software v2.0. For CAPS markers, PCR products were digested with the corresponding restriction enzymes (*Ssp I*, *Apa I*, or *BsrB I*; Table A4) under the conditions recommended by the manufacturer and separated on 1.5% agarose gels.

### 4.5. Breeding Strategies and Selection Procedures

Conventional pedigree breeding and a multi-parent double top cross (DTC) scheme were used to introduce TTM-derived material into improved wheat backgrounds. In the pedigree scheme, the white-grained, high-GP cultivar AK58 was crossed with TTM. From the F_2_ generation onward, the *TaMyb10-D1*-linked KASP marker was used to track the TTM-type marker genotype, while phenotypic selection was applied for plant height, plant architecture, grain color, and germination performance. Plants carrying the TTM-type marker genotype and showing desirable agronomic phenotypes were self-pollinated and advanced through successive generations. Marker-assisted and phenotypic selection were repeated from F_2_ to F_5_ until homozygous, phenotypically stable lines were obtained. For multi-parent breeding, a DTC scheme [32] was used to combine TTM-derived material with complementary agronomic traits from CM104, SM753, and SM580: CM104 × TTM × SM753 × SM580 / SM580. In the F_2_ and F_3_ generations, field selection focused on stripe rust resistance under natural infection, reduced plant height, and plant architecture. In the F_4_ generation, the *TaMyb10-D1*-linked KASP marker was used together with visual selection for white grain and laboratory measurement of germination percentage. Plants meeting the combined genotypic and phenotypic selection criteria were advanced.

### 4.6. Genetic and Statistical Analyses

Analyses were performed on three segregating populations described in Section 4.1. Grain-color segregation was analyzed in the F_2_ population and derived F_3_ families of TTM × CS and in the F_2_ population of TTM × AS661634. The observed numbers of red- and white-grained individuals were tested for goodness-of-fit to the expected Mendelian ratios using chi-square (χ^2^) tests. The χ^2^ value was calculated as χ^2^ = Σ[(O-E)^2^/E], where O and E are the observed and expected numbers, respectively. The degrees of freedom (df) were calculated as the number of phenotypic classes minus one. A *p*-value > 0.05 was considered indicative of a good fit to the tested genetic model. These χ^2^ goodness-of-fit tests were performed using the built-in function in Microsoft Excel.

Marker–trait association was further evaluated in the TTM × AK58 F_2_ and F_3_ segregating populations. A total of 77 F_2_ lines and 92 F_3_ lines were evaluated for germination percentage (GP) and genotyped using the *TaMyb10-D1*-linked KASP marker targeting SNP 3D_572163094. For each generation, lines from the two extremes of the GP distribution were selected for comparison: the lowest 10% constituted the low-GP group, whereas the highest 10% constituted the high-GP group. The F_2_ low- and high-GP groups each contained 7 lines, and the corresponding F_3_ groups each contained 9 lines. The frequency of the TTM-type marker genotype in each group was calculated as the proportion of lines carrying this marker genotype. Fisher’s exact test was used to evaluate whether the frequency of the TTM-type marker genotype differed between the low- and high-GP groups.

Statistical analysis for quantitative traits, including GP, plant height, thousand-kernel weight, and kernels per spike, was conducted to compare means between parental lines and derived breeding populations. For comparisons among multiple groups (e.g., parental lines and F_1_ hybrids), one-way analysis of variance (ANOVA) was performed, followed by Tukey’s honest significant difference (HSD) post hoc test for mean separation when a significant F-value (*p* < 0.05) was obtained. All quantitative data are presented as mean ± standard deviation (SD). These analyses were performed using IBM SPSS Statistics software (version 25.0).

## Figures and Tables

**Table 1:** Grain color and germination percentages of TTM and selected wheat cultivars across environments.

Accession Name	Seed Color	GP_2022 Wenjiang (%)	GP_2022 Chongzhou (%)	GP_2025 Wenjiang (%)
TTM	White	40.63 ± 9.38 b	57.09 ± 19.45 b	12.67 ± 3.06 d
AK58	White	98.72 ± 1.88 a	95.74 ± 9.52 a	98.67 ± 2.31 a
CM104	Red	53.90 ± 6.63 b	82.47 ± 6.16 a	53.33 ± 1.15 c
CS	Red	20.58 ± 12.28 c	41.82 ± 4.69 b	16.67 ± 4.16 d
CY21	White	97.00 ± 1.03 a	92.23 ± 1.76 a	94.96 ± 1.06 a
CY23	White	93.18 ± 7.56 a	93.86 ± 12.57 a	86.00 ± 2.00 b
CY25	White	98.80 ± 0.88 a	96.94 ± 1.06 a	95.01 ± 1.14 a
SM580	White	99.60 ± 0.89 a	99.57 ± 0.95 a	100.00 ± 0.00 a
SM753	White	95.99 ± 3.99 a	99.53 ± 1.04 a	100.00 ± 0.00 a
MY1848	White	99.79 ± 0.47 a	99.80 ± 0.45 a	99.67 ± 0.58 a

Note: GP, germination percentage. Data are presented as mean ± standard deviation from three replicate Petri dishes, each containing 50 seeds (n = 3). Different lowercase letters within a column indicate significant differences (one-way ANOVA followed by Tukey’s HSD, *p* < 0.05).

## Data Availability

All raw data involved in this study are available from the corresponding author upon reasonable request.

## Appendix A

**Table A1:** Grain color phenotypes of F_1_ plants from crosses between the white-grained landrace TTM and red- or white-grained elite wheat cultivars.

Cross Combination	Generation	Red	White
Red-grained parents (crosses)			
TTM × AS661634	F_1_	All	0
TTM × CS	F_1_	All	0
CS × TTM	F_1_	All	0
TTM × CM104	F_1_	All	0
CM104 × TTM	F_1_	All	0
White-grained parents (crosses)			
TTM × AK58	F_1_	0	All
CY21 × TTM	F_1_	0	All
CY23 × TTM	F_1_	0	All
TTM × CY23	F_1_	0	All
CY25 × TTM	F_1_	0	All
SM580 × TTM	F_1_	0	All
TTM × SM580	F_1_	0	All
TTM × SM753	F_1_	0	All
TTM × MY1848	F_1_	0	All

Note: “All” indicates that all observed plants/seeds showed the respective grain color; “0” indicates none.

**Table A2:** Segregation analysis of grain color in populations derived from crosses between TTM and red-grained cultivars CS and AS661634.

Cross	Generation	Observed Phenotype (R:W)	Expected Ratio (R:W)	χ^2^ Value	*p* Value
TTM × CS	F_2_	118:46	3:1	0.813	>0.05
TTM × CS	F_3_	108:56	5:3	0.787	>0.05
TTM × AS661634	F_2_	147:7	15:1	0.764	>0.05
TTM × AS661634	F_2_	147:7	3:1	34.36	<0.01

Note: Degrees of freedom (df) = 1 for all tests. R:W = red-grained:white-grained plants.

**Table A3:** Sequence polymorphisms selected for molecular marker development.

ID	Annotation	Chr.	POS	CS	TTM
*TraesCS2D03G1100600*	*F3H*	Chr2D	593890141	C	T
*TraesCS3B03G0942000*	*C4H*	Chr3B	603799446	A	G
*TraesCS4B03G0926600*	*CHS*	Chr4B	646788223	C	A
*TraesCS4B03G0902300*	*FLS*	Chr4B	639499163	G	C
*TraesCS4A03G0505800*	*WD40*	4A	465755349	T	C
*TraesCS3D03G1031600*	*MYB10*	3D	572163094	G	A

Note: Chr., chromosome; POS, physical position; *F3H*, flavanone 3-hydroxylase; *C4H*, cinnamate 4-hydroxylase; *CHS*, chalcone synthase; *FLS*, flavonol synthase; *MYB10*, R2R3-MYB transcription factor; *WD40*, WD40-repeat protein.

**Table A4:** Primer sequences used for marker assays.

Gene	Marker Type	Primer	Sequence	Enzyme
*C4H*	KASP	*C4H*-COM	AGGTGCCACTCTGAAAACCC	NA
		*C4H*-FAM	GAAGGTGACCAAGTTCATGCTCTTGGCGTCGTGGAGGTTCA	
		*C4H*-HEX	GAAGGTCGGAGTCAACGGATTCTTGGCGTCGTGGAGGTTCG	
*F3H*	KASP	*F3H*-COM2	CGACCGAGCTTCATCAAGGAA	NA
		*F3H*-FAM	GAAGGTGACCAAGTTCATGCTCGTCGCCTCCCCCCAGGC	
		*F3H*-HEX	GAAGGTCGGAGTCAACGGATTCGTCGCCTCCCCCCAGGT	
*FLS*	KASP	*FLS*-COM	CTCACCATCCTGATGATGGAC	NA
		*FLS*-FAM	GAAGGTGACCAAGTTCATGCTGTATGTACTTGGTGCATCACC	
		*FLS*-HEX	GAAGGTCGGAGTCAACGGATTGTATGTACTTGGTGCATCACG	
*Myb10*	KASP	*MYB10*-COM	CTGCTGGTTCCTCTTGCT	NA
		*MYB10*-FAM	GAAGGTGACCAAGTTCATGCTGTGGTCGCTGATCGCGGG	
		*MYB10*-HEX	GAAGGTCGGAGTCAACGGATTGTGGTCGCTGATCGCGGA	
*WD40*	CAPS	*WD40*-F1	GGGGCAAATCTTCAGGAGCA	*Ssp* *I*
		*WD40*-R1	GTCCCGTCTTCACCCTTGTA	
		*WD40*-F2	TATCCAGAGGACATAGGAG	
		*WD40*-R2	ACCCCAATTCCATCATTTAC	
*CHS*	CAPS	*CHS*-F	ACAGATTCTCTTGAGCTTCTCCT	*Apa* *I*
		*CHS*-R	AGTTTGCCTCCATGTTCTCAAC	
*Myb10*	CAPS	*Myb10*-F	TGATGATGACGTGCATTAATTCAC	*BsrB* *I*
		*Myb10*-R	CTGCTGGTTCCTCTTGCT	

Note: For *WD40*, the nested PCR amplification method was adopted. Firstly, primers F1 and R1 were used to amplify Product 1. Then, using Product 1 as a template, primers F2 and R2 were employed to amplify Product 2, followed by restriction enzyme digestion.

**Table A5:** Statistical analysis of linkage and association between linked marker and grain color in the TTM × CS F_2_ population.

Linked Marker	Max LOD Score	χ^2^ Value (Independence Test)	df	*p*-Value	Genetic Inference
*TaMyb10-D1*	>45	64.00	1	<0.0001	Significantly linked.
*TaC4H-B1*	0.008	0.57	2	0.751	Not linked.
*TaFLS-B1*	0.006	0.94	2	0.624	Not linked.
*TaF3H-D1*	0.004	2.14	2	0.343	Not linked.
*TaWD40-A1*	0.003	1.23	2	0.540	Not linked.
*TaCHS-B1*	0.002	0.08	2	0.962	Not linked.

**Table A6:** Germination percentage and agronomic performance of TTM, parental cultivars, and advanced breeding lines.

Line	GP (%)	Plant Height (cm)	TKW (g)	Kernels per Spike
TTM	12.67 ± 3.06 d	135.0 ± 5.0 a	40.67 ± 1.25 d	70.0 ± 3.6 a
AK58	98.67 ± 2.31 a	72.0 ± 3.0 c	58.54 ± 1.86 b	52.0 ± 4.2 b
CM104	53.33 ± 1.15 b	91.0 ± 4.0 b	65.14 ± 2.03 a	72.0 ± 5.1 a
SM753	100.00 ± 0.00 a	90.0 ± 3.5 b	60.11 ± 1.94 b	70.0 ± 4.8 a
SM580	100.00 ± 0.00 a	83.0 ± 4.0 b	65.95 ± 2.21 a	54.0 ± 3.9 b
SMBK4	46.93 ± 4.73 b	75.0 ± 3.0 c	38.17 ± 1.42 d	54.0 ± 4.1 b
SMBK3	29.33 ± 3.06 c	89.0 ± 4.5 b	49.44 ± 1.58 c	78.0 ± 5.3 a

Note: GP, germination percentage; TKW, thousand-kernel weight. Data are presented as mean ± standard deviation. For GP, three replicate Petri dishes containing 50 seeds each were evaluated (n = 3). Plant height, TKW, and kernels per spike were measured using three independent plants (n = 3). Different lowercase letters within each column indicate significant differences among lines (one-way ANOVA followed by Tukey’s HSD test, *p* < 0.05).

**Table A7:** Germination percentage (GP) of SMBK3 lines grouped by *TaMyb10* and *PHS1* haplotypes.

Haplotype Combination	Number of Lines	Mean GP (%)
*TaMyb10* (+) + *PHS1* (+)	11	27.43
*TaMyb10* (−) + *PHS1* (+)	5	41.66
*TaMyb10* (+) + *PHS1* (−)	1	40.59
*TaMyb10* (−) + *PHS1* (−)	2	52.87

Note: For *TaMyb10-D1*, + and − indicate presence or absence of the TTM-type marker genotype, respectively; for *PHS1*, + and − indicate presence or absence of the reported resistance haplotype.
